# Design of emotional branding communication model based on system dynamics in social media environment and its influence on new product sales

**DOI:** 10.3389/fpsyg.2022.959986

**Published:** 2022-08-02

**Authors:** Yin Zhang, Zhongfang Tu, Wenting Zhao, Lu He

**Affiliations:** Hainan Normal University, Haikou, China

**Keywords:** social media, emotional marketing, sales of new products, brand emotion, brand design, system dynamics

## Abstract

In the current social media environment, emotional branding communication has become a common marketing tool for brand owners, and therefore it has become particularly important and urgent to study it. Based on the perspective of brand equity theory, combined with the new characteristics of marketing communication in the social media environment, this paper constructed an emotional branding communication model in the social media environment. The system dynamics (SD) method was used to simulate and analyze the new product marketing system to assess whether it could stir the emotional needs of the consumers and resonate within their hearts. This paper discusses the asymmetric communication of different brands regarding the same commodity to determine the impact of this exchange mechanism, that is, only the weak brands in the market initially adopt marketing methods, while the strong brands do not participate in social marketing activities. It was found that the influence of marketing frequency and marketing intensity on symmetric and asymmetric communications was different. In the face of different types of competitors, the marketing strategy of weak brands needs emphasis. Through unit consistency test, structure verification test, effectiveness, and rationality test, it was proven that the emotional branding communication model and new product sales interaction simulation model established in this paper were reasonable and effective.

## Introduction

In the increasingly fierce competitive market environment, possessing an excellent brand value becomes an important determining factor in the success or failure of an enterprise. A good brand can build on customer preferences and attract more brand loyalists. However, the establishment of brand loyalty is closely related to psychological factors of consumers to a large extent, in addition to excellent product quality, perfect market adaptability of the product, and promotional marketing strategies. Emotional marketing is focused on feelings and it builds customers' loyalty to the brand based on emotion. It does this by meeting the emotional needs of the customers, providing them with a psychological connection and preference for the product, and forming a loyal customer base that does not buy from any other enterprise or brand. With the development of computer, network, and communication technologies, social media, such as Weibo and WeChat, are being used by enterprises as marketing tools in the development of their advertising business. In the process of enterprise development, whoever can identify customers' needs and provide them at the lowest cost with the most satisfactory service in the shortest time will win the competition. As an important part of technological innovation, the coordination between product innovation and technological innovation plays an important role in improving the technological innovation capability of manufacturing enterprises. The coordinated development of product innovation and process innovation is an effective way to improve the technological innovation capability of manufacturing enterprises.

The term emotional branding refers to placing consumers' emotional peculiarities and needs at the core of brand marketing strategy and realizing the business objectives of enterprises using emotional marketing and advertising (He et al., 2016). Against this background, a vast majority of internet users have gradually turned into consumers or potential consumers with consumption goals and willingness. Their purchasing power and attention have become indispensable scarce resources for brand marketing activities, and this has come to be termed the “attention economy” (Myfanwy et al., 2012). Once consumers develop brand emotion, they will subconsciously have the same affection for other products from the same brand. The degree of affection can not only make consumers continue spending on the product, but also reduce their price sensitivity. At the same time, it produces subsequent chain reactions such as brand trust and brand loyalty. When there is no special innovation in products or services, emotion becomes the key factor for consumers to make purchasing decisions (Mingione et al., 2019). Emotional marketing captures the special emotional needs of consumers, implements the emotional marketing strategy of enterprises, and runs the main thread of “emotion” through the whole process of marketing activities.

Social media platforms have gathered a large number of users because of their low threshold and easy operation. People's ways and channels of obtaining information through social media have changed. At the same time, it has accelerated the interaction between marketers and users, and users gradually accept, like, and get used to exchanging information with brands through social media. In this study, the dissemination of news and opinions is integrated, and the mode and mechanism of its comprehensive dissemination are explored by computer simulation (Ha and Perks, 2010). In addition to “news dissemination,” which obviously belongs to the dissemination of information category, the dissemination of ideas and opinions also falls under the same category. Therefore, this paper sums up its research by discussing the coupling dynamic process of emotional branding communication in the social media environment. A successful emotional marketing strategy can not only impress consumers by releasing the brand's core emotional energy in the process of product marketing but also subtly strengthen consumers' brand awareness and establish a good brand image in the consumers' minds.

The innovative contribution of this paper lies in the establishment of a coupling dynamic model of emotional branding communication in the social media environment. It is found that marketing frequency and intensity have different effects on symmetric and asymmetric communications. Facing different types of competitors, there is a need to emphasize the marketing strategy of weak brands. Through unit consistency test, structure verification test, and effectiveness and rationality test, it is proved that the emotional branding communication model and new product sales interactive simulation model established in this paper are reasonable and effective.

The paper is structured in five parts. The first part describes the background of emotional branding communication. The second part expounds on the relevant research results of emotional marketing. The third part describes the modeling basis for the research method and analyzes the SD model construction and the design of the emotional branding communication model in the social media environment. The fourth part describes the results and analyzes the influencing factors for stability. It includes an analysis of network word-of-mouth propagation and coupling dynamics. Finally, the full text is summarized in the last part.

## Related work

### Research on brand emotional marketing

The concept of social media came into being in the early 21st century. Social media is a new online media that provides users with participation and space. This kind of media has the characteristics of openness, participation, dialogue, community, and communication. Emotional marketing communication has become a common marketing tool for brand owners in the current social media environment. It has therefore become particularly important and urgent to study emotional marketing communication in the social media environment.

Parent and Séguin (2010) hold that social media is a media system in which people generate or obtain the content they need through a decentralized human-based network. It allows people to form various forms of relationships based on personal, political, and commercial applications in the online world. Yang et al. (2015) saw social media marketing as a practice based on social media, which could simplify the form of dialogue and realize content sharing among enterprises, influencers, information seekers, and consumers. Morgan-Thomas and Veloutsou (2013) discuss the concept and significance of advertising brand communication from the concept and significance of the brand and explain the contents of advertising brand communication through six aspects, such as brand name, visual symbol, auditory symbol, core concept, cultural connotation, and brand image. They also detail a few basic laws of advertising brand communication. Hamzah et al. (2014) hold that the new media characterized by interactivity and autonomy makes advertisers' autonomous communications quite successful, while at the same time severely challenging the core elements of traditional advertising such as “payment,” “identification,” and “impersonal communication.” In a sense, “integrated marketing communication” has resulted in the elimination of advertising noumenon, and “brand communication” has become the inevitable trend in advertising evolution.

Duan et al. (2021) argue that the key to integrated marketing lies in maximizing the influence of communication through the communication methods that all target audiences may accept. According to Kuesten (2011), from the perspective of brand communication, experiential marketing takes brand communication closer to the consumers and guides consumer behavior by satisfying their emotional and aesthetic experiences. Consumers' true feelings about brands come from experience. Through experiential marketing, the added value of brands can be better spread. Church (2013) explain that brand association refers to the cognitive and emotional connection between consumers and brands, and this kind of brand association will cause consumers to allocate resources reasonably and then influence their consumption behaviors. With the deepening of consumers' cognitive and emotional brand association, consumers' behaviors will show stronger support for brands. Paladino and Pandit (2012) carried out an in-depth analysis of emotional marketing by combining theoretical analysis with practical examples. They believed that emotional marketing was the inevitable trend of the future development of marketing and the core value of marketing. The essence of emotional marketing was to impress consumers and build their brand loyalty. Lao (2016) explain the influence of emotional factors on consumers' purchasing decisions and the overall influence of emotional factors on emotional marketing.

Scholars have different perspectives on how to undertake emotional marketing. After a comprehensive analysis of some scholars' views, emotional marketing can be summarized into four aspects. First, the development of emotional products, emphasizing the consumers' emotional and psychological needs and packaged under the premise of the product use value. Second, the utilization of emotional trademarks to attract and impress consumers with true feelings and kindness when designing trademarks. Third, determination of the emotional price, and finally consolidation of the relationship between the brand and consumers to establish their brand loyalty.

### SD related research

System dynamics (SD) describes interrelated systems with causality diagrams and stock first-class diagrams and quantifies the dynamic characteristics of simulation systems with simulation language. Among them, the stock represents the state of system variables, and the variables at different time points are different with each state. The flow chart represents the activities of system variables, such as the consumption of inventory, and the employment or dismissal of personnel. With the development and perfection of SD, systematic thinking has gradually formed a series of important principles and has become an effective tool to study and deal with social and economic complex system problems.

Ricardo Saavedra et al. (2018) define SD as an applied discipline based on the system feedback control theory that uses computer simulation technology as the main means to quantitatively study the dynamic behavior of system development, which is a branch of system science. The particularity of SD makes it suitable for dealing with long-term and periodic problems, research on insufficient data, complex socio-economic problems with low requirements, and complex time-varying systems with non-linear and multiple feedbacks. It can do long-term, dynamic, and strategic simulation analysis and research, and analyze the structure and dynamic behavior of the system (Ding et al., 2018). According to Wong et al. (2022), the profit level of service product supply enterprises is determined by currency voting. To maximize profits, the main body of an enterprise obtains customer value by continuously improving its quality of products and services, to constantly develop its core competitiveness. Kochan et al. (2018) proposed a comparative marketing system and argued that marketing was comparable. In their work, Cheng et al. (2018) establish the dynamic model of the global electronic communication demand system and study the negative feedback relationship of demand using SD. Tan et al. (2018) comprehensively discuss the theory and method of SD and establish various SD models, which have been widely acknowledged in academic circles and enterprises. Scholars have advanced the research on the theory and practical application of SD, and the research results are very rich.

As for brand acceptance, existing research mostly regards it as a pure viewpoint in the dynamics process. However, if the brand acceptance dynamics process is only regarded as the application research of viewpoint dynamics, the influence of social marketing information dissemination on the inherent image of the group is ignored. Hong et al. (2018) proposed an information dissemination model with infection probability. In the model, individuals were divided into an unknown state, known state, and immune state. A communication model of two kinds of competing information (information 1 and information 2) in a unified network was proposed. The results show that when the degree of nodes in the network is large enough or there are large clusters in the network, information 1 will occupy an absolutely dominant position in the network. Cai and Liang (2021) discuss the influence of individuals' cognition of danger on the transmission rate in multi-coupled networks. The results show that the network structure has a significant relationship with disease control, and the greater the difference between network layers, the more difficult it is to control the disease. Furthermore, they approximated the simulation results using mean-field analysis and obtained the threshold by the self-organizing method.

## Research method

### Foundation of modeling

For the realistic problem of brand acceptance in social marketing information dissemination, the existing research mostly simplifies it into the interactive process of views among groups. However, in the process of consumption, brand buyers will encounter a large volume of promotional information related to brand marketing activities, and consumers will be affected by competing marketing information. It is significant to research this interactive mechanism of brand communication in the social media environment, which we do in this study. First, it provides the main path of the communication process, and second, it emphasizes the status of transformation between receivers in different communication environments and modes. In the era of social media, it is necessary to re-examine the position between the receiver and the sender, as well as the differences in various elements of communication in different technical backgrounds. Due to the double impact of globalization and pan media, communication has changed from being a professional activity by professional institutions to the daily activities of all social organizations and members. At the macro level, it shows obvious integrity characteristics, and at the micro level, it shows multiple differentiation trends. From the perspective of integrity, China's international communication needs to adhere to the concept of system and make major adjustments and systematic innovations in macro strategy, overall layout, systems, and mechanisms. Through effective integration and coordination, the overall efficiency of the system can be maximized. From the perspective of differentiation, international communication should implement accurate communication guided by efficiency. Through the careful selection of various transmission resources and means, the transmission effect of different lines can be maximized. A good grasp of integrity and differentiation is conducive to building an international communication model with multiple objectives, diverse forms, and different objects.

**Figure 1:**
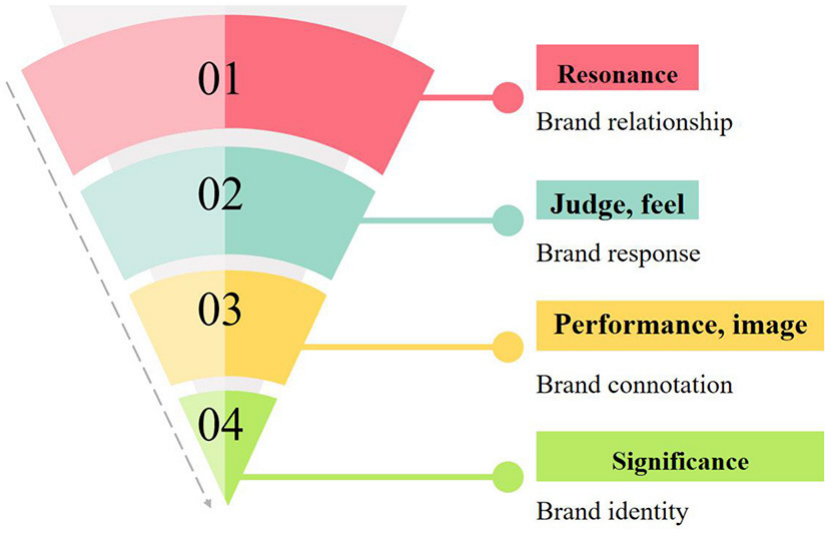
Consumer-based brand equity model.

The content function value, content entertainment value, social interaction value, and brand interaction value of brand fan pages positively influence the click intensity and comment participation (Nyadzayo et al., 2020). In social media, trust and interpersonal factors have a significant positive impact on users' participation in online word-of-mouth communication. In this marketing campaign, users have completely become creators of content and participants of publicity. By perfectly integrating marketing content with consumer interaction, brands have enabled more users to have emotional resonance and brand goodwill at the subconscious level, effectively improving user attachment, and brand loyalty. Kuesten (2011) proposed that the successful model in the field of brand management is based on the brand equity model of consumers, as shown in Figure 1.

This model shows that the establishment of a strong brand usually needs four consecutive steps: building a clear brand identity, creating appropriate brand connotation, guiding the correct brand response, and creating an appropriate brand relationship with consumers. On the left side is the rational route for consumers to recognize the brand, and on the right side is the emotional route for consumers to recognize the brand. Rational combination with the perceptual route can achieve the highest stage of brand equity “resonance.”

In the absence of external disturbance, the acceptance of a certain brand can be simply understood as a problem of viewpoint dynamics. Some people in the group approve of the brand, while others do not, and individuals influence each other, which will eventually lead to a distribution of the product's approval. In the propagation dynamics model selected in this paper, individuals can only be in two states: the unknown state and the known state. One of the basic assumptions of the model is that the process of information dissemination usually occurs among individuals with the same views with high probability, that is, individuals with the same original ideas are always more willing to accept information and disseminate it but will happen among individuals with different views with low probability.

**Figure 2:**
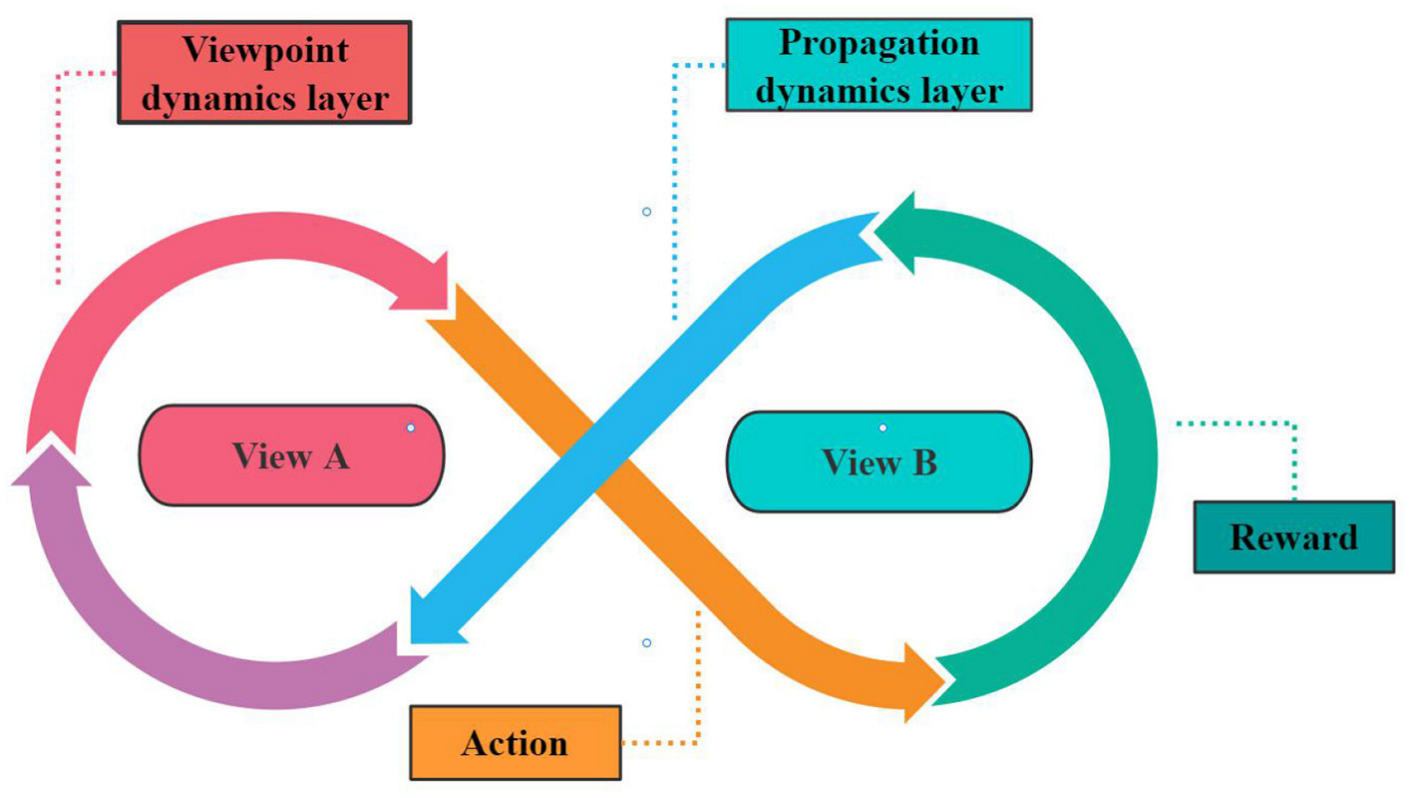
Schematic diagram of coupling model.

This article discusses the situation of different market shares in the market at the beginning, and how weak brands with a small market share can still defeat the dominant competitors through social marketing. For instance, A and B, respectively, indicate two competing brands for a certain product and reaching out to consumers through social marketing activities. Figure 2 shows the whole coupling dynamic process which is produced by the interaction of two dynamic types.

With the advancement in Internet technology, continuous popularization of smartphones, and increasing popularity of new social media—including its advantages of high transparency, interactive participation, and sociability—social media has attracted more users to use it to publish, share and disseminate information or obtain resources. The relationships that people build in virtual networks are also spreading rapidly. The growing popularity of this new way of communication also provides unlimited opportunities for enterprises to carry out innovative marketing activities. This paper focuses on a coupling dynamic model of the interaction between opinion and information dissemination. The dynamic process of the influence of social media on brand acceptance in the rule network is studied by using the agent-based computer simulation modeling method. When the system is stable, the time τ it takes to explore the stable state of the system, and the spread range ρ_∞_(*A*) of marketing information of weak brands, for the proportion of *A*(*I*_*A*_) individuals in the final state of the system is explored in this paper.

The performance *P* value of the system is a measure of brand effect, is defined, and is expressed by formula (1) among all the experimental times, the proportion of the experimental times that the system finally agrees with the weak brand *A* viewpoint:


(1) $$\begin{array}{r} P=\frac{\sum_{\rho_{\infty }}\left(A\right)=1}{experiments} \end{array}$$

The shopping environment is the comprehensive embodiment of the brand's overall image, the most direct place for consumers to buy goods and services, and the important frontier for contact with customers. Emotional marketing. also referred to as sensory marketing, engages sight, hearing, touch, and smell. SD can handle such social and economic systems well. In this paper, we establish an SD model that observes the behavior of the system through the model, seeks the improvement of the system, and proposes solutions to problems.

The goal of our research on the marketing system is to develop an appropriate strategy to promote the long-term growth of sales and ensure that the marketing system produces significant and lasting improvement.

### SD model construction

SD model is a system dynamics model, which analyzes the dynamic and dialectical relationship between information feedback, system structure, function, and behavior space. It is a bridge for understanding problems between systems and communicating natural and social sciences. Based on system theory, information theory, cybernetics, and computer technology, it reflects the dynamic mechanism of the actual system according to the system state, control, and information feedback. This is achieved through the establishment of a simulation model, with the help of a computer simulation test in a scientific method.

System dynamics uses the system science idea that “every system must have a structure, and the system structure determines the system function.” According to the feedback characteristics that the internal components of the system cause and affect each other, the root cause of the problem is found in the internal structure of the system, instead of using external interference or random events to explain the behavior of the system.

Marketing theory provides a theoretical foundation for the marketing of the new product sales market. Marketing refers to a social and management process in which individuals and collectives create, offer, sell, and freely exchange products and values with others to obtain what they want. When the marketing demand is absent, the task of the marketer is to link the advantages of products with people's natural needs and interests, to stimulate this demand. When the demand declines, the marketer needs to analyze the reasons for the decline, and then adopt creative product re-marketing, stimulate demand, and reverse the downward trend.

A unique brand needs to create an unforgettable experience for its consumers and provide them with a kind of ethereal pleasure while conveying brand connotation, to deepen consumers' brand perception. The purpose of experience design is to stir consumers' resonance and change their perception so that the experience satisfies meeting consumers' higher psychological needs.

**Figure 3:**
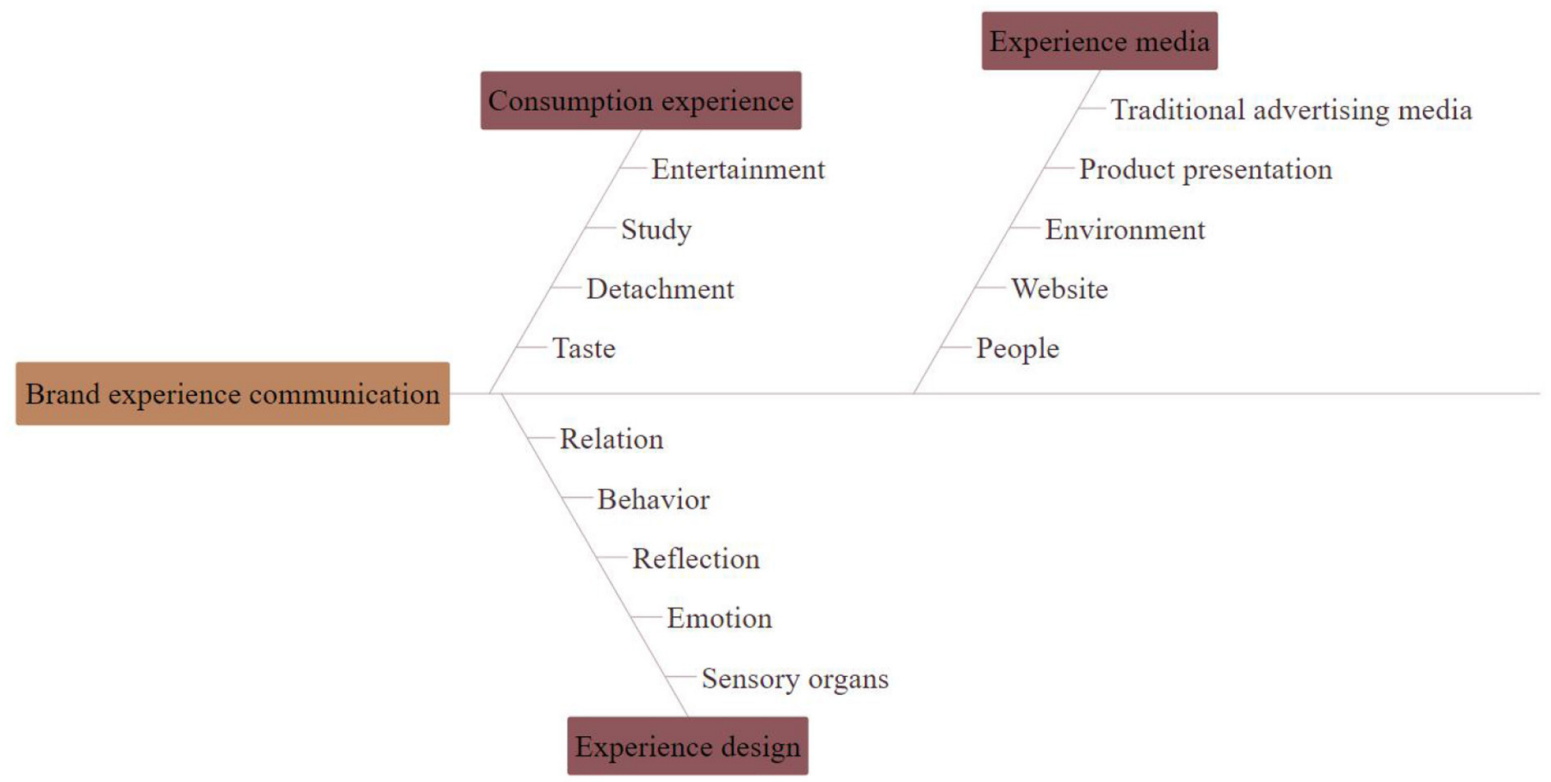
Brand communication mode.

Brand experience is the cumulative result of brand-related events that consumers have participated in Lao (2016). Generally speaking, the purpose of brand experience is to establish a good image of the enterprise in consumers' minds by meeting their higher value needs. The communication model of experience includes experience design, experience media, and consumption experience, as shown in Figure 3.

The information output under new media has changed from one-way communication to interactive communication, from one-to-one to one-to-many, which determines that brand marketing communication under new media is interactive, giving consumers more opportunities to participate and express, and the disseminator and receiver of information are not fixed; consumers are also realizing value co-creation with brands in the process of deepening brand identity. This is the creative application of emotional marketing strategy in Internet communication, and it is also the interactive layout of brand building and the interactive dialogue space between consumers. At the same time, the application effect of strategies also directly affects consumers' emotional attachment and loyalty to brands. Because of its interactive and interesting features, it has become an explosive marketing product. Strengthening cognition in the connection of strong audience relationship resonates with emotional interaction. Brands also occupy the propaganda high ground in marketing communication by creating unique social codes.

In the revenue subsystem, with the improvement of customers' lifetime value, the assets of enterprises will increase, and more resources will be invested in the improvement of enterprises' products and services, which will further improve customers' satisfaction and loyalty, thus guiding customers' consumption and enhancing customers' lifetime value. Sales volume will become one of the variables that would be of more concern, and it would also be the most important indicator to measure the quality of the system. The issue is how to solve the problem of sales volume in real enterprises while meeting the overall goal of the enterprise. There is a need, therefore, to develop a strategy for long-term sales growth. The marketing activities of enterprises, a factor that has nothing to do with the product itself, will trigger the “happy” mood of consumers, and this consumer mood will also have a certain impact on brand loyalty.

Product innovation and process innovation can improve the economic benefits of manufacturing enterprises. However, because manufacturing enterprises are in a complex market environment, and their situations vary widely, manufacturing enterprises choose or focus on product innovation and process innovation.

Because the demand for life-cycle products is growing faster and more capricious, companies are faced with more shortage risks in the early stage, which may bring false demand and reduce the number of potential customers. As a result, companies may have to invest in a large inventory but still lose some sales. When consumers have strong brand feelings or enjoy a high-quality brand experience, the level of brand commitment will improve. Consumers who have positive feelings for the brand will have full confidence in the brand, which will make them less susceptible to the negative events of the brand or the marketing tactics of competitors.

According to the theory of social influence, three main factors influence the formation of individual views: the individual's views, the views of neighboring groups, and the influence of government policies and mass media. This paper selected the voter model based on the majority principle. The viewpoint interaction model constructed in this paper uses $O={⋃}_{i=1}^{N}o_{i}$ to represent the viewpoint set. A discrete value, such as 1 or 0, was used to express the individual's opinion of supporting or opposing the brand. The exchange rules of views in the model are shown in formula (2):


(2) $$\begin{array}{l} \{\begin{array}{cc} O_{i,t+1} & =\text{sgn}\left(\phi *O_{i,t}+\left(1-\phi \right)*\text{sgn}\left(\sum_{j}O_{j,t}-6-0.6\right)\right)\\ \text{sgn}\left(x\right) & =\{\begin{array}{c} 1,x\geq 0\\ 0,x<0 \end{array} \end{array} \end{array}$$

*O*_*i,t*_ represents the viewpoint of an individual *i* at time, and *O*_*i,t*+1_ is the viewpoint of the individual *i* the following time. A two-dimensional grid with a periodic boundary in the Moore neighborhood was selected as the network environment of the model. The viewpoint of the current individual *i* in the model is determined by two parts. The first part is determined by the individual's last-held viewpoint; the second part is determined by eight neighboring individuals in the Moore neighborhood of the individual *i*.

**Figure 4:**
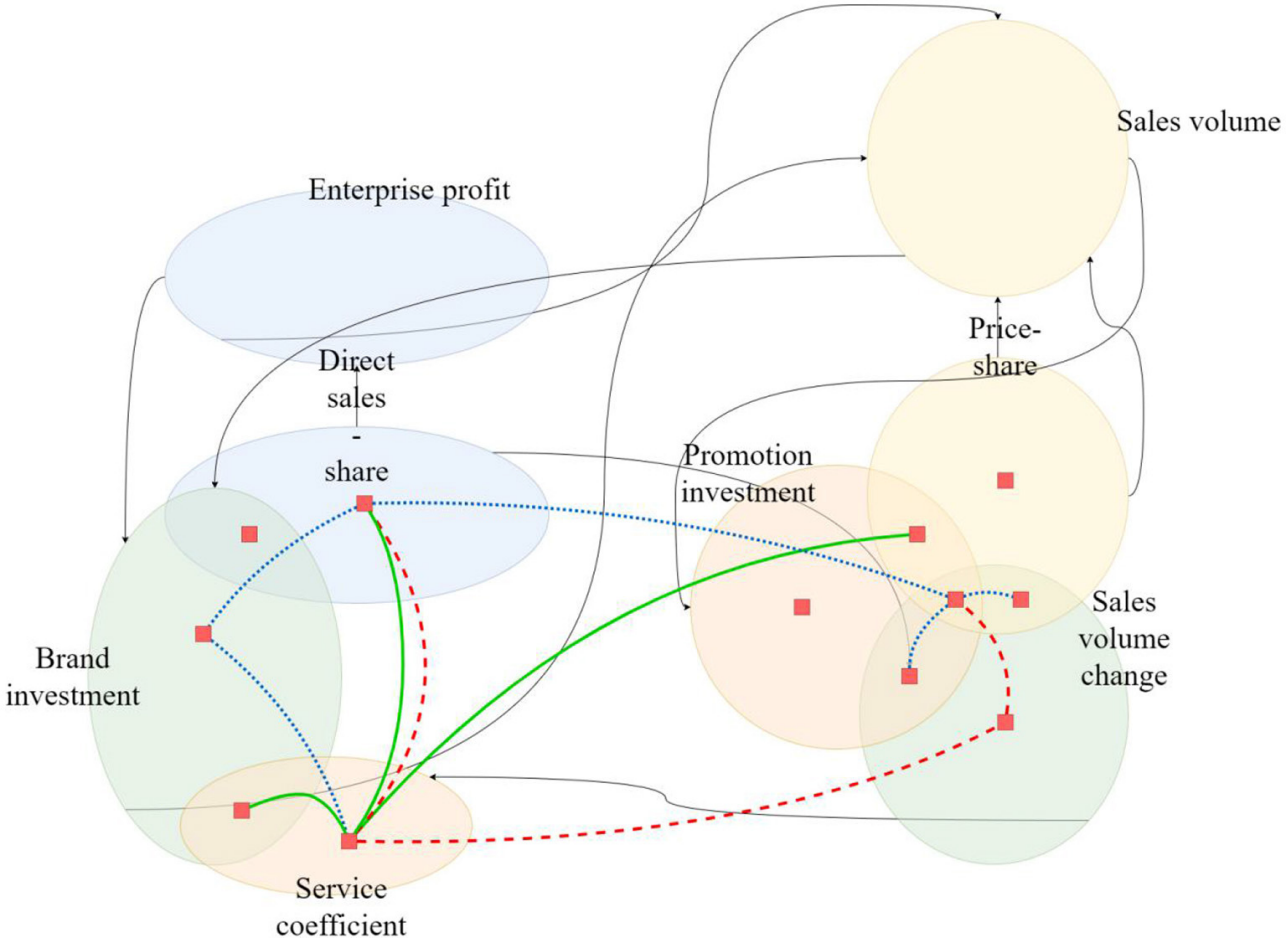
Overall flow chart of the marketing system.

There are five main variables in Vensim, including state variables, rate variables, auxiliary variables, constants, and exogenous variables. Determining variables mainly includes analyzing the variables of the system and the relationship between variables, defining variables (including constants), and determining the types and main quantities of variables. Through the above causal loop analysis, the main factors affecting the system are determined. Then, all subsystems are combined to form the overall flow chart of the marketing system, as shown in Figure 4.

Between the revenue and cost subsystems and with an increase in customers' income, enterprises have more resources to invest in customer service and product research and development, attracting more customers to become corporate customers. On the other hand, the enterprise's investment increases the customers' expectations, which leads enterprises to invest more resources to maintain customers' expectations. If the customers' expectations cannot be met, customers will be lost, thus reducing the enterprise's customer income.

**Figure 5:**
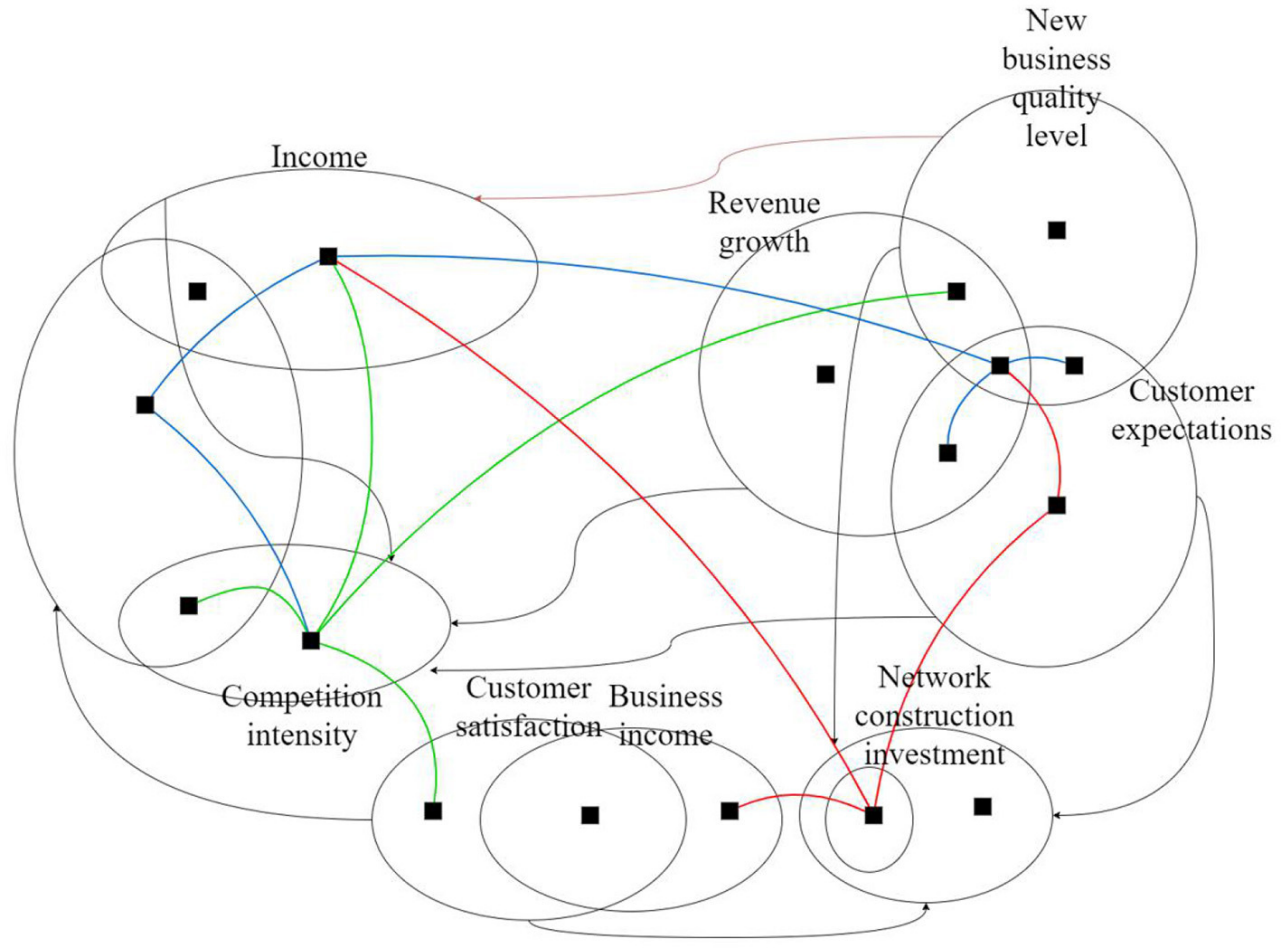
Income subsystem flow diagram.

When enterprises join the ranks of competing for high-value customers, the competitive factors will reduce the customer transfer cost, shorten the customer life cycle, and increase the income, which will enable enterprises to attract additional new customers, thus further increasing the customer life cycle length of enterprises. The flow chart of the income subsystem, developed after analysis, is shown in Figure 5.

The enterprise technological innovation system, as the integration of enterprise technological innovation-related activities, has systematic characteristics. It is composed of an organizational system, rule system, resource allocation system, and decision-making system. The flow variable of this subsystem is income, where the income is brought by customers to the enterprise, and the flow rate is the increase in income. When the whole enterprise develops well, it has more resources to invest in the operation of the enterprise, which can ensure that the enterprise maintains a high level of service quality, and at the same time can invest more resources in the development and promotion of data and value-added services, thus improving new services and customer satisfaction. When the customer lifetime value of an enterprise increases, the assets of the enterprise increase by a large margin, thus ensuring that the enterprise can invest more resources in its daily operations. However, an increase in the investment cost to the enterprise leads to an increase in customer cost, which leads to a decrease in customer lifetime value and a consequential decrease in enterprise profits.

This paper holds that the interactive relationship between product innovation and technological innovation of manufacturing enterprises, especially the relationship between competition and cooperation, is mainly reflected in the decision-making process and manufacturing process of technological innovation of manufacturing enterprises. An increase in investment in one subsystem will lead to a decrease in investment in another subsystem. In the manufacturing process, the technological innovation subsystem carries out its activities influenced by the output factor, product complexity, product-process correlation, and its own system operation in the product innovation subsystem. The dynamic evolution of the interactive relationship between product innovation and process innovation is beneficial to the improvement of manufacturing enterprises' own technological innovation capability (product innovation capability and process innovation capability), and ultimately improve the independent innovation capability of manufacturing enterprises.

**Figure 6:**
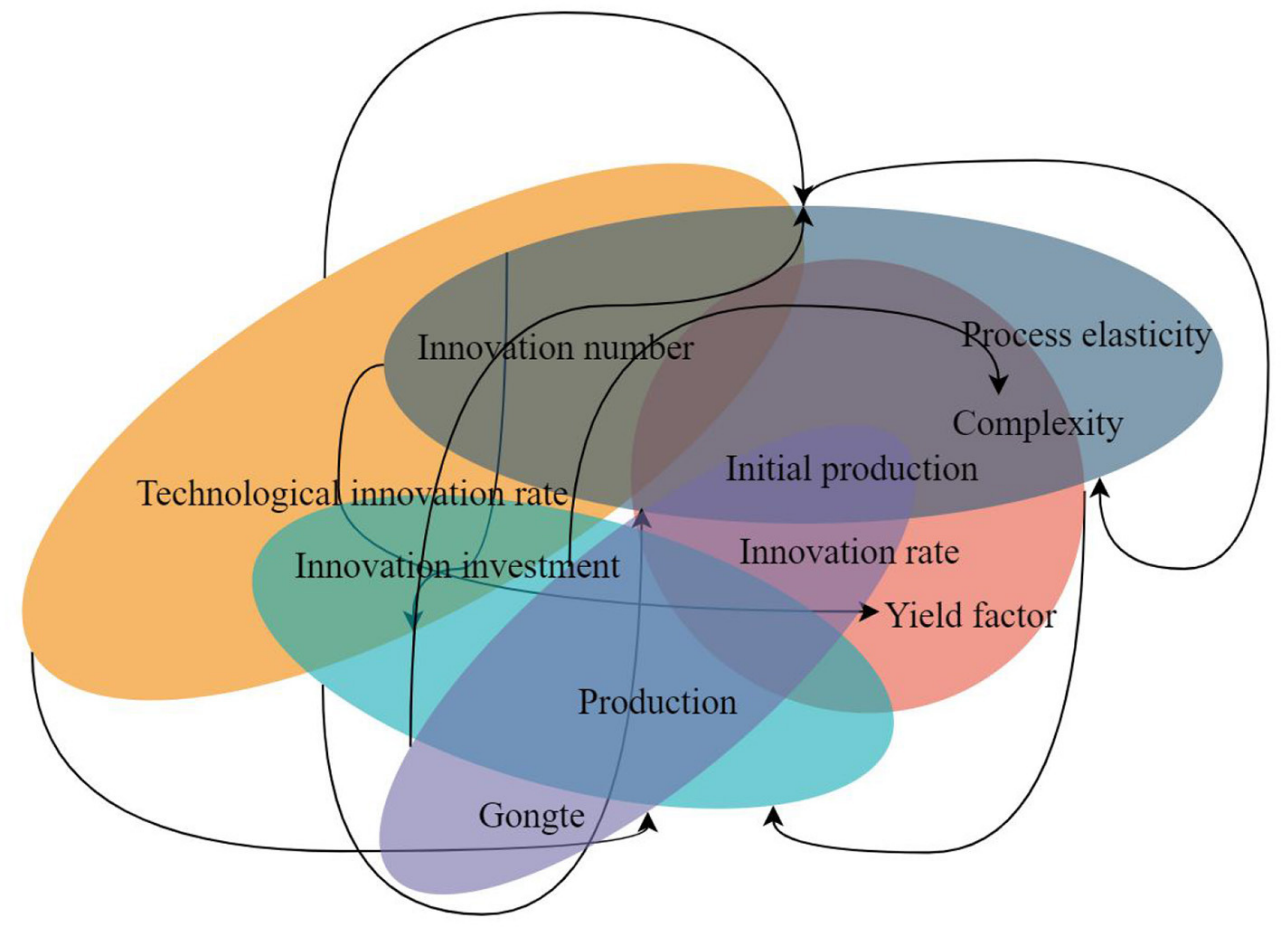
Causality diagram of the interaction between product innovation and process innovation.

Based on the analysis of the whole system and the interaction between product innovation and process innovation of manufacturing enterprises, the system causality diagram is shown in Figure 6.

In the SD equation of the interaction between product innovation and process innovation, we determined the horizontal variables as product innovation number and process innovation number; the rate variables were product innovation rate and process innovation rate, while other variables were auxiliary variables and constants. Whether the manufacturer could directly obtain market information had a great influence on the final factory inventory. If the market information could be obtained directly, likewise the manufacturer could also obtain the maximum total profit of the system. Therefore, compared to long-life products, short-life products need to consider the market exit time of their products, and different market exit times can offer different total profits to the supply chain system. If the total profit of the system needs to be maximized, then those managing the supply chain need to consider the time when the products will exit the market.

According to SD theory, although the function of a complex system depends on the elements of the system, it depends more on the relationship between these elements. If only the elements are analyzed by ignoring the relationships among them (i.e., input, output, and causality), the essential characteristics of the system cannot be understood.

**Figure 7:**
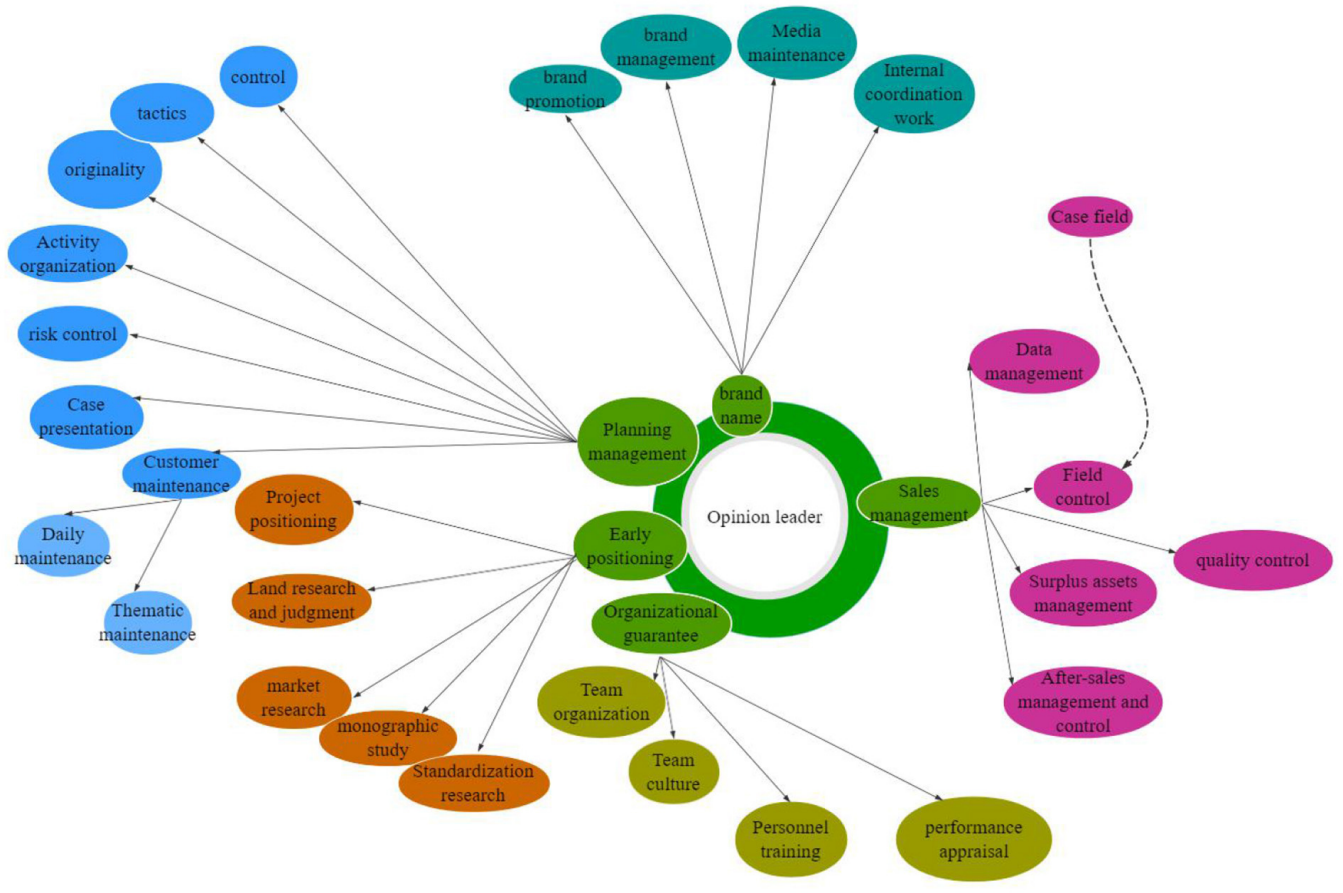
Causality diagram of online word-of-mouth communication.

The impact of influencers on online word-of-mouth communication is divided into two parts and four ways. The positive part is “professional ability,” “social activity ability,” and “relationship coordination ability,” while the negative part is “information interaction power.” The study of the causal relationship between the influencing factors in the process of online word-of-mouth communication is shown in Figure 7.

The threshold value of transfer indicates that the network word-of-mouth disseminators need to keep confidential the valuable information they own. When the information levels of both the network word-of-mouth disseminators and the receivers are closed, the information transfer will no longer occur. The “professional ability,” “social activity ability,” and “relationship coordination ability” of influencers have a positive correlation with the information transfer mechanism, while “information interaction power” has a negative correlation; the information integration and absorption ability is higher than that of the receiver. This is because the recipient's main goal is to obtain information from the Internet word-of-mouth communicators and absorb and transform the received information, and therefore the recipient's desire to absorb information is higher than the other party's.

The relationship between the factors influencing the stability of technological innovation alliance of complex product systems is complicated and constantly changing. Hence, the influencing factors are typically a large-scale system with multivariable, high-order, and non-linear dynamic feedback (Kuesten, 2011). If mobile advertising information is valuable to consumers' friends or relatives in some way, it will be a good reason for them to forward it. This demonstrates that users' perceived value of use positively affects their willingness to disseminate the information.

In social media, the altruistic motivation and self-improvement of the target audience positively and significantly influence the goals of viral marketing and communication (Mingione et al., 2019). Enterprises launch marketing activities online through network communication, and it becomes a powerful marketing strategy to communicate with consumers through word of mouth (Parent and Séguin, 2010).

#### Design of emotional branding communication model for social media

**Figure 8:**
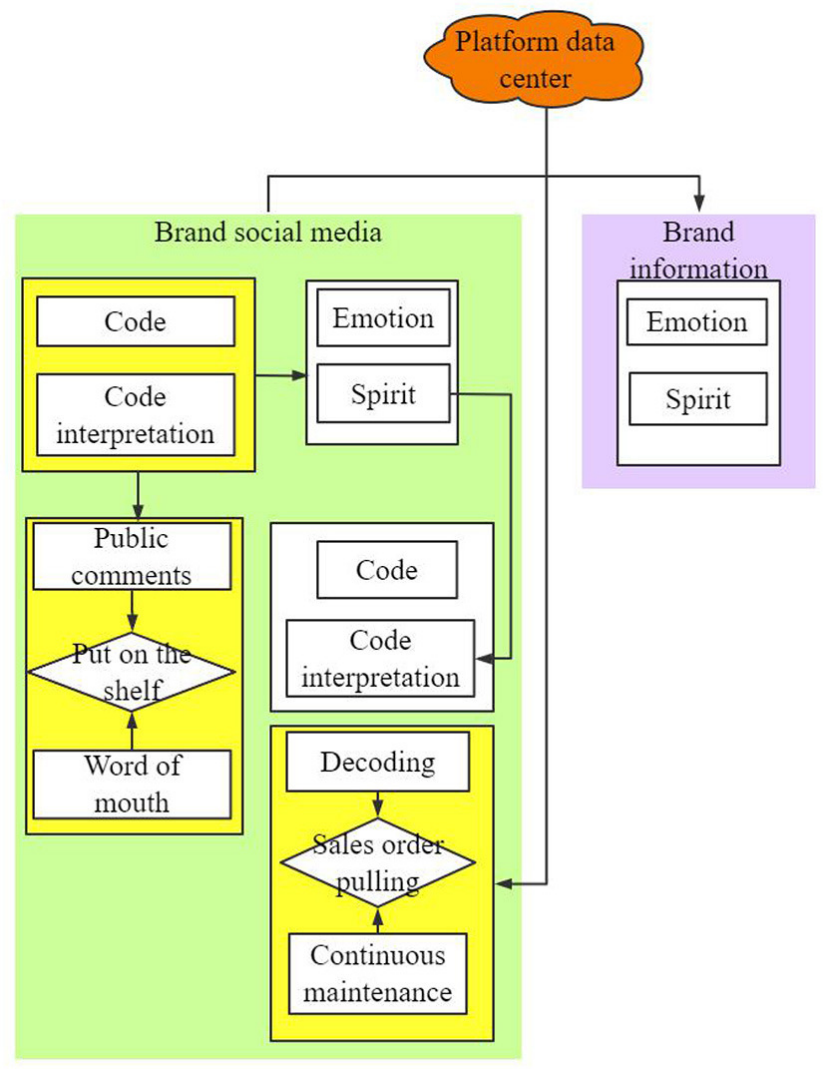
Marketing communication model of brand emotion in a social media environment.

The essence of emotional branding communication is that enterprises convey the marketing information with emotional value to consumers through targeted communication channels. The aim is to let consumers experience particular emotions after receiving the information, so that consumers and brands have psychological resonance, thus achieving the purpose of marketing communication. Based on a comprehensive review of research on the mode of interactive communication, combined with the data and research in previous sections, the process of social media brand communication was developed (refer to Figure 8).

This process diagram of brand interactive communication is based on the social media environment and for use on the social media platform. In the process of interaction, the interaction subjects are brand and social media users. Brands are mainly spread by building social media matrices that are usually distributed, centralized, centrally radiating, multi-central radiating, and integral. In this mechanism diagram (Figure 8), the relationship between transmission and reception of interactive subjects is not obvious, i.e., the brand and users are both communicators and audiences, exchanging information, correcting, and influencing each other through the interaction. As a result, the subject of communication achieved equality in communication status. Therefore, in the interactive communication between the brand and the users, this study determined that the communication process of both parties is cyclic and continuous when coding and decoding.

The remarkable achievement of marketing communication in the social media is that the traditional one-way linear structure between the disseminator and the information receiver is broken. Everyone can transmit their opinion through the social media platform, and users also have the initiative to receive, pay attention to and share information. Brands can create informative content that reflects product performance and brand values, or convey emotional information to users through social media communication channels. In turn, the users, through their inherent impression and judgment of the brand and their emotional response to the brand's emotional messaging, develop a subconscious selection mechanism about the brand. Direct emotional expression and communication, however, should avoid obvious information and theme expression, underestimating the understanding ability of users, or interference of other environmental factors, resulting in the brand failing to communicate effectively with users through emotional marketing communication.

The speed of capacity expansion increases the investment cost and production capacity in the operation of the supply chain. However, there will be a time delay because manufacturers need to purchase equipment and raw materials to expand their capacity. People from a certain milieu know about the environment and other people in it, and at the same time, ensure that their behavior conforms to the social norms in their environment. However, online, people can exist in different milieus at the same time, such as enjoying purchasing on the shopping platform while learning English on the online education platform. Situations can be tailored to provide more immersive experiences for the audience through technical channels.

The inherent interactive nature of the Internet makes it a natural medium for emotional branding. The active assembly of emotional communities and emotional cultural groups makes the boundaries between situations increasingly blurred. The emotional marketing of brands using the Internet should actively create situations and emotional visualizations when promoting brand information and selling brand products online. With the gradual penetration of the Internet into people's everyday life, the integrity of traditional social relations, consumption concepts, and market structure has disintegrated. Differentiated demands have led the network media platform to form various communities. To make communication more effective, attention must be paid to subdivisions within groups. Therefore, if brands want to innovate marketing strategies, they should also focus their efforts on the channel side, so that their content is inclusive, reflects users' reality, and enables a deep connection between users and the advertised scenarios (He et al., 2016).

## Result analysis

### Analysis of influencing factors of stability

In the social media environment, users are no longer passive receivers but actively participate in the communication. The social media communication process is different from the traditional process. The biggest difference lies in the increased initiative of the users. Emotional marketing communication content, a special kind of information content, is easy to evoke people's emotional resonance and psychological identification because of its perceptual nature. Enriched with obvious interactive characteristics, social media has emerged as a common manifestation of information processing where users re-create and re-disseminate information content.

**Figure 9:**
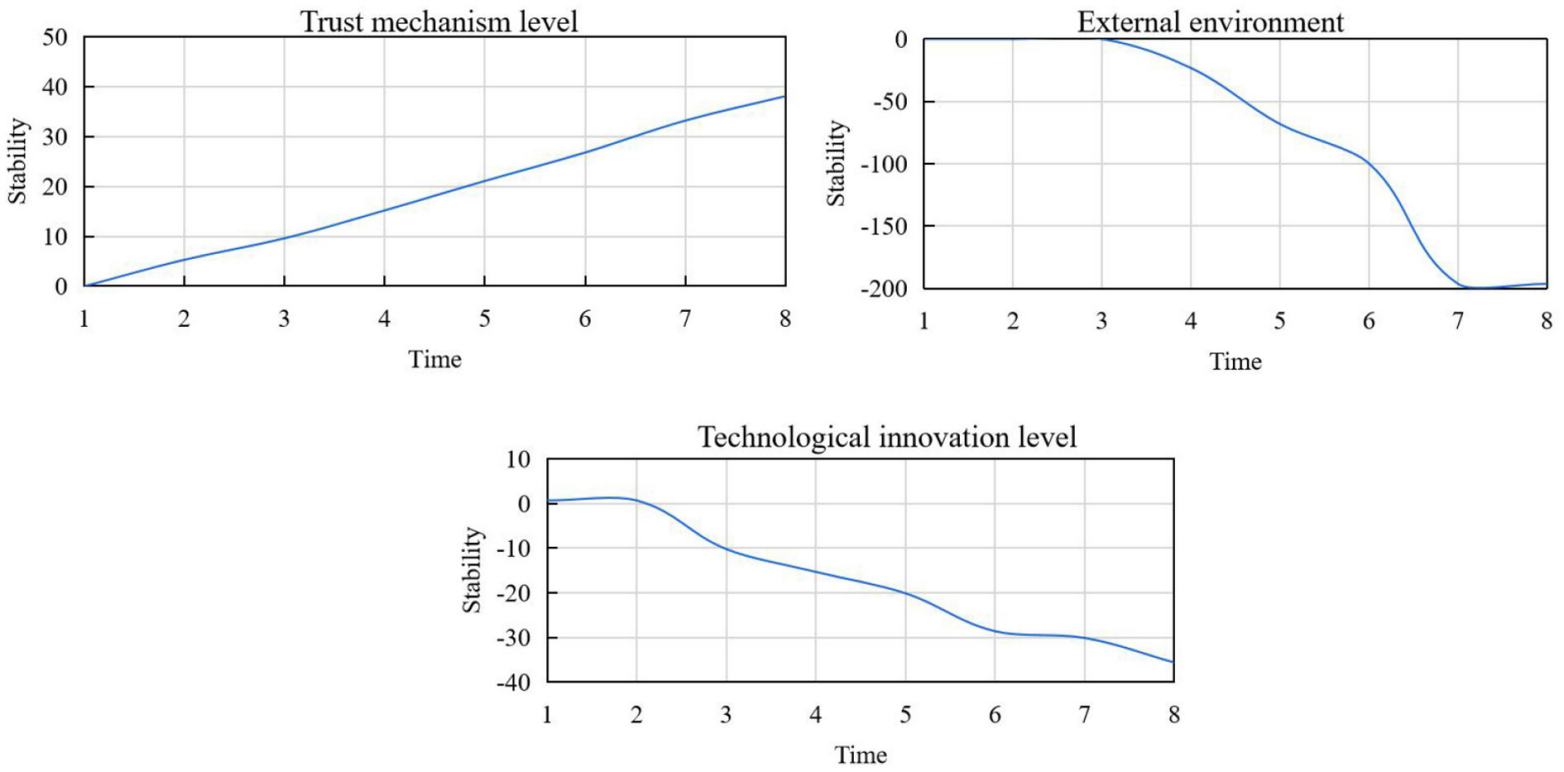
State variable influence curve.

Using attitude change theory, this study examined the marketing information characteristics of social media as the stimulus to explore if it can bring about change in users' attitudes. The stimuli were categorized under three dimensions, namely content perceived value, information source influence, and usage perceived value. The simulation period was 8 months with the time step at 1, mutual influence coefficients among the factors at 0.6, the function coefficients of the related factors at 1.6 or−1.6, and the pulsation numbers of the related factors starting from 0, with an interval time of 6 months. The result obtained after simulation by Vensim software is shown in Figure 9.

The simulation parameters selected in this paper pertain to only one case. When the simulation parameters change, the curve changed correspondingly, but the basic curve trend remained the same. We can draw the following conclusions from this analysis. At the initial stage of alliance construction, the trust mechanism of the alliance was not perfect, and because of the low level of technological innovation, the level of the complex product project itself was at the lowest level. With the development of the alliance, the level of trust mechanism and technological innovation increased, and with it, the level of the project improved continuously. With the development of the alliance, complex products met the needs of consumers. With an increase in demand, opportunism among alliance members emerges, resulting in the gradual decline of alliance cooperation and alliance stability.

In this scenario, the weekly order quantity not only considers the average weekly sales volume but also considers the influence of other factors. To meet product demand, manufacturers must monitor the planned production and increase production capacity accordingly. When the production capacity is improved, output increases and the under-ordered quantity of the manufacturer decreases, as well as the expected in-transit order quantity. The main loop represented by the thick line is expanded to include the seller's order quantity and inventory, which is also a negative feedback loop, making the causal relationship of the system more complicated and closer to reality.

According to the emotional marketing communication model under the social media environment constructed in this paper, emotional information content needs to reflect product information and brand values at the same time, which are two important aspects of emotional content, both of which are indispensable. The connotation of the emotional information conveyed by the brand to the users not only contains “performance,” that is, the functional performance of the product but also contains a unified “image” composed of its brand values and legacy. Once the values of emotional information deviate, it will directly affect the information connotation of brand owners in emotional marketing communication and cause adverse consequences to marketing communication effect and strategy.

### Analysis of network word-of-mouth communication

The information gap between communicators and receivers in online word-of-mouth communication is widening with time. This is because communicators have strong information integration and absorption ability, and they can integrate and absorb new information and increase their information while spreading it word-of-mouth. Emotional marketing communication is a communication process in which brands influence users' emotions through a series of activities. Emotion, as a cognitive experience, is highly subjective and easily influenced by the outside world.

It is therefore important to add heart-warming or pleasant content to the information so that users experience happiness when reading the content. Promotional activities or activities related to corporate brands should be initiated so that consumers can participate in them, circulate the details, or comment on social media to achieve the psychological needs of brand interaction and social interaction.

**Figure 10:**
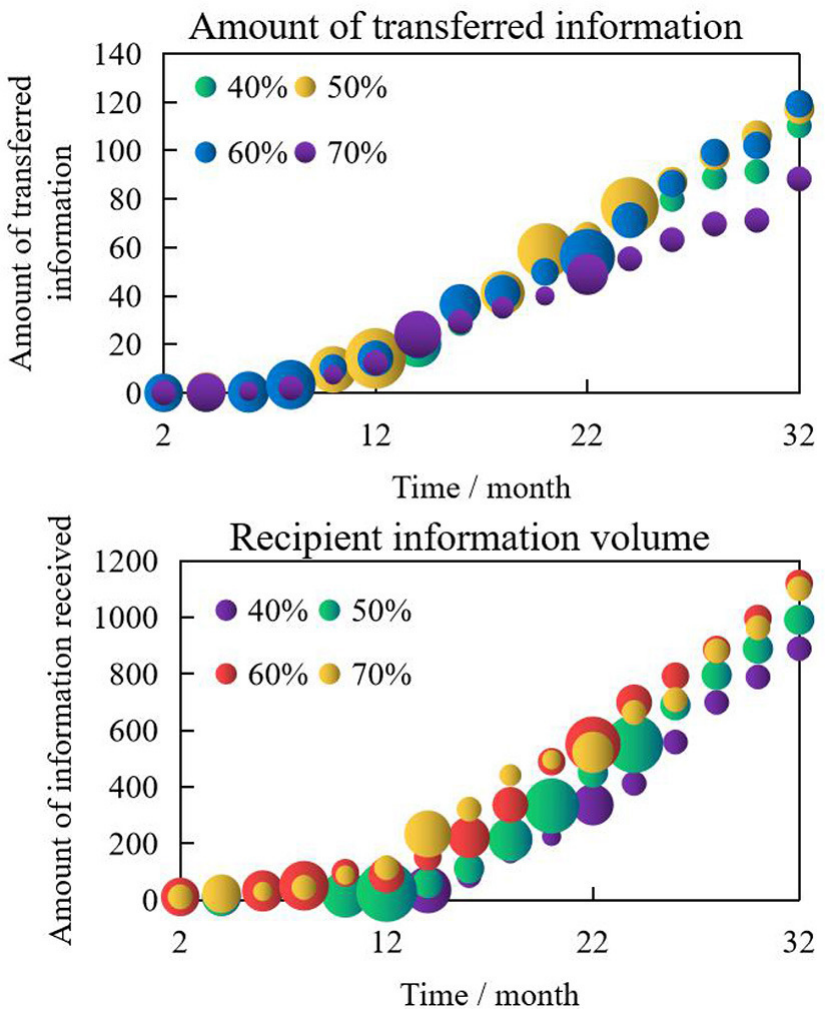
Sensitivity analysis of influencers' power to online word-of-mouth communication.

Among the four factors that affect the amount of information transfer, the sensitivity of the transfer mechanism was analyzed by changing the parameters of influencers in the model to determine the level of influence they had on online word-of-mouth communications. We gradually increased their level of influence, in order of 40, 50, 60, and 70% gtt four schemes. The sensitivity analysis of influencers' power to online word-of-mouth communication is shown in Figure 10.

The power of influencers on the volume of online information transferred through word-of-mouth communication was the same as that of transferring information directly. This shows that the relationship between influencers and the volume of transferred information first increases and then decreases, showing a “parabolic” relationship. Reasonably using the positive power that influencers wield by providing them with relevant information for quick word-of-mouth transmission, and tapping into their professional ability, social activity ability, and relationship coordination ability, along with reducing the purchase cost for the recipients receiving the promotional information will effectively promote online word-of-mouth communication.

Before the Internet and social media technology, consumers' demands could not be effectively expressed as the user feedback mechanism was very imperfect. It was collected through manual sampling surveys, telephone complaints, written complaints, and other channels. However, these methods had poor timeliness and limited scope, and brands could not respond immediately and quickly. To some extent, the research on the interactive mechanism diagram of brand communication is helpful for brands to express their value proposition, maximize the effect of brand communication, upgrade the quality of products and services, and improve their market competitiveness. It also plays a certain role for users to express their demands, exchange their consumption experiences, and meet their own needs.

Taking content perceived value, information source influence, and perceived use value as independent variables and behavior attitude as dependent variables, a multiple regression analysis was conducted. The results are shown in Table 1.

**Table 1:** Regression result.

**Variable**	**β**	***t***	**Sig**.
Content perceived value	0.155	2.441	0.012
Influence information source	0.303	4.563	0
Perceived value of use	0.386	7.214	0
*F*	190.25		
*R^2^*	0.661		

The regression equation of social media advertising marketing information characteristics to behavior attitude was significant (*R*^2^ = 0.661). The significance of the perceived value of use, the influence of information sources, and the perceived value of content were all <0.05, which indicates that these three variables have a significant influence on behavior and attitude.

Much of the above-mentioned research on information dissemination is mainly aimed at a single object. However, in many realistic situations, the spread of relevant news and the diffusion and evolution of public ideas are intertwined and influence each other, forming a coupling process of various kinds of communication. The importance of marketing frequency is gradually revealed, which shows that in the “independent” market environment if the competitors adopt strong marketing strategies, it will have a better effect on increasing marketing frequency. The marketing strategy of “single point and high frequency” allows weaker brands to have a higher chance to beat their competitors and occupy a larger market share.

An enterprise's profits, minus its cost and deduction of corresponding taxes and fees are consistent with the actual system. Among them, the cost is affected by unit cost and sales volume, while the income is affected by sales volume and product pricing. Taxes and corporate profits form a feedback loop, and income tax and management expenses are deducted according to a certain percentage of profits. When the pricing of an enterprise is lower than the average market price, the sales volume of the enterprise increases greatly, but the corresponding cost is also very high, which lowers the overall profit level. The larger the sales volume, the lower the profit, and even the loss. Although this strategy is stable, it does not have the advantage of long-term development, has no positive upward trend, and is easily threatened by other risks in the market.

### Coupling dynamics analysis

Considering that there are many competing brands for certain kinds of goods in the market, brands often adopt different marketing strategies for competitors. For example, new brands in the market tend to make greater efforts, comparatively, to enter the market. When enterprises provide high-quality products and services, they must be supported by corresponding funds, talents, and other resources to ensure that the quality and value proposition of products and services are consistent. Brand values are an important part of business assets that enable consumers to identify and remember the brand's interests and personality and are the main force driving consumers to recognize, like, and even fall in love with the brand.

The change in consumer behavior patterns in the network environment has led to new consumer contact points. Media is no longer limited to the original fixed form, and different media types need to be integrated and disseminated. All feasible contact points between consumers and products or brands need accurate brand prediction, and real-time information communication with consumers at every possible extrusion point. Social media occupies an important position in the field of online media. The characteristics of huge user groups, information fission mode of communication, various virtual community social relationships, and instant information release mode all make social media the mainstream market of brand marketing communication. Cross-domain cooperation entails integrating online and offline marketing resources to achieve better emotional marketing and publicity impact. Cross-brand cooperation entails a strong brand alliance, attracting loyal fans of both sides, and exerting greater emotional influence.

**Figure 11:**
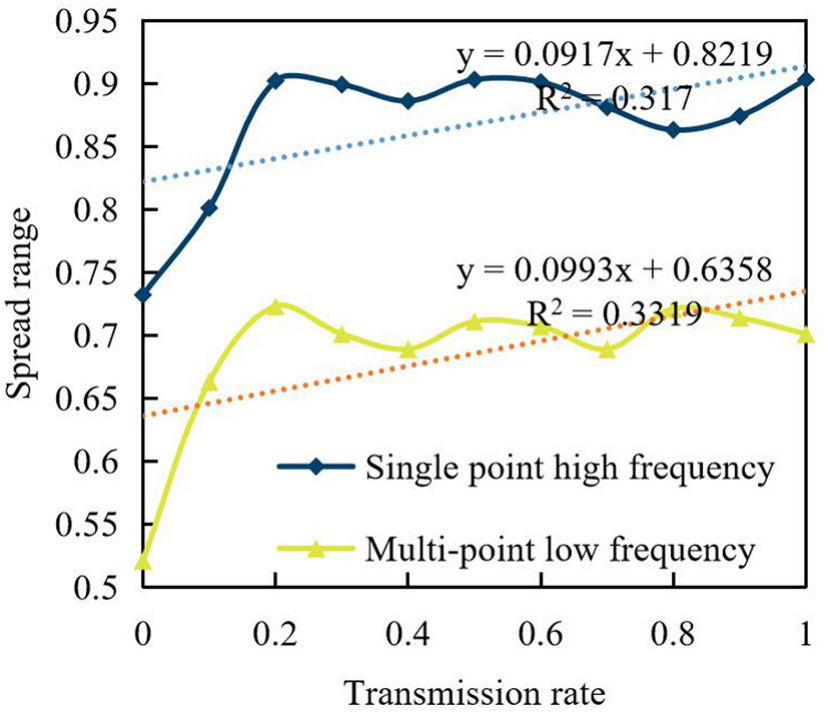
Influence of propagation rate on coupling dynamics.

Figure 11 shows the influence of the propagation rate on the system *P* value, the propagation range, and the time step taken for the system to reach the steady state when the propagation frequency *f* = 0.002, 0.02, 0.2 is selected.

It can be found that, under different propagation frequencies *f*, the *P* value of the coupled dynamics system under symmetric propagation has no obvious and stable change, which indicates that the increase in propagation frequency has no obvious promoting effect on the consensus of coupled dynamics under symmetric propagation.

Two brands adopt the same social marketing strategy: “single-point high frequency” or “multi-point low frequency.” In the coupled dynamics model, the interaction between viewpoint dynamics and propagation dynamics, as well as the similarities and differences between coupled dynamics and single dynamics process under the action of different propagation frequencies and transmission rates, are mainly analyzed from the information propagation range, the *P* value of the system and the consumption time step of the system to reach steady state. The germination of users' awareness of interaction and multi-channel information exchange among users not only require brands to be more cautious and realistic in making promises, introducing product functions, and providing after-sales service, but also require them to continuously follow up interactive services, such as timely response and real response.

Among all the interactive forms, comments are the most direct way to express users' views. Comments from 76 social media users were characterized by noise and fragmentation. Most social media platforms do not set up a screening mechanism for users' comments, allowing users to freely post comments; so it is easy to generate meaningless symbols or comments. Besides getting to know the brand information through free channels, the interviewees believe that other potential consumers can also see their opinion on the products or enterprises, thereby encouraging enterprises to maintain their existing service levels through positive evaluation while at the same time, drawing the attention of potential consumers and enterprises in case of a negative evaluation. Through suggestions and opinions, obtained by consumers' direct communication with brands, enterprises can produce products according to users' needs or improve through after-sales service.

**Figure 12:**
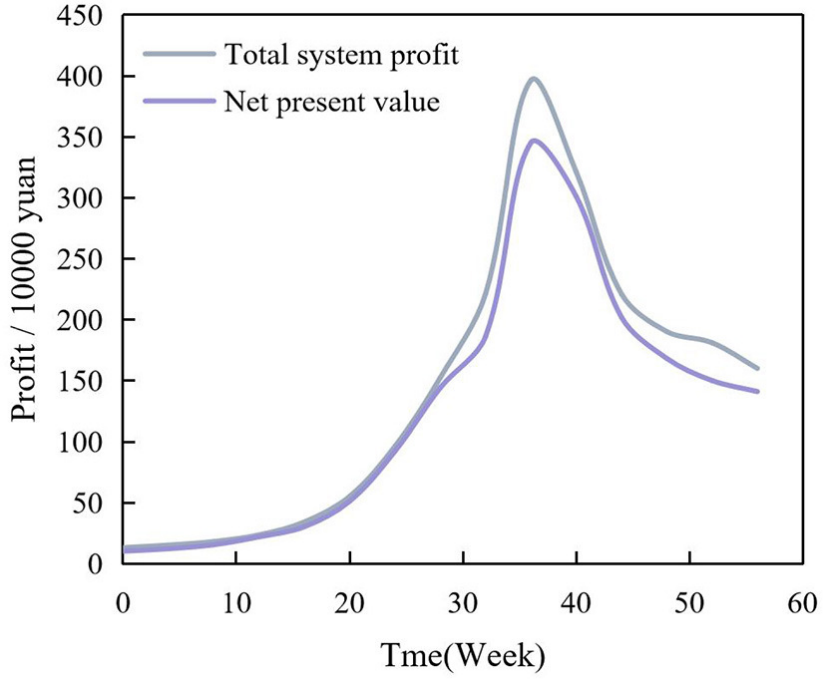
Total profit curve of supply chain.

The total profit of the supply chain is the main index to evaluate the performance of the supply chain. Because the simulation period of the supply chain in this study was more than 1 year, to accurately describe the total profit of the system brought by the product supply chain in operation, we used the net present value to describe the total profit. The discount rate of the net present value was set at 10%. Figure 12 shows the total profit curve of the supply chain system.

Through the analysis of the total profit curve and simulation data of the supply chain system, it can be concluded that the total profit value of the supply chain does not always increase in the whole operation cycle. Therefore, in the operation of supply chain, to get the maximum profit during the operation of the supply chain, the products can be withdrawn midway and not essentially wait until the end of the operating cycle or the end of the product life.

### Sales analysis of new products

Because of diverse consumer demands, as well as the restricted product life cycle, and technological innovations and renewal, the development of new products require constant attention by enterprises. For the design and production of new products, the foothold lies in the enterprise itself, i.e., before the products enter the market. In marketing, competitors can be divided into four levels: desire competition, category competition, product form competition, and brand competition. Given that the impact of the initial market sales pricing of new products is long-term and extensive, it needs to be considered from a strategic viewpoint. For pioneering new products, two strategies can be adopted during the initial launch period: skimming pricing or penetration pricing. For imitating new products, it is necessary to choose according to the quality, price, and target market of competitors' products.

On the other hand, increasing the investment in product and process innovation subsystems at the same time promotes the innovation activities in both the subsystems, leading to their successful completion. Therefore, manufacturers should focus on avoiding the negative emotions of consumers, to improve the satisfaction level as much as possible, reduce the adverse effects of negative word of mouth on enterprises, and finally improve brand loyalty using the influence of brand trust and emotion. In addition, optimism and excitement have a great influence on post-purchase behavior. They are also two positive emotions that can't be ignored. Surprises through sales promotion activities or elegant gifts could be planned to make consumers excited or optimistic.

**Figure 13:**
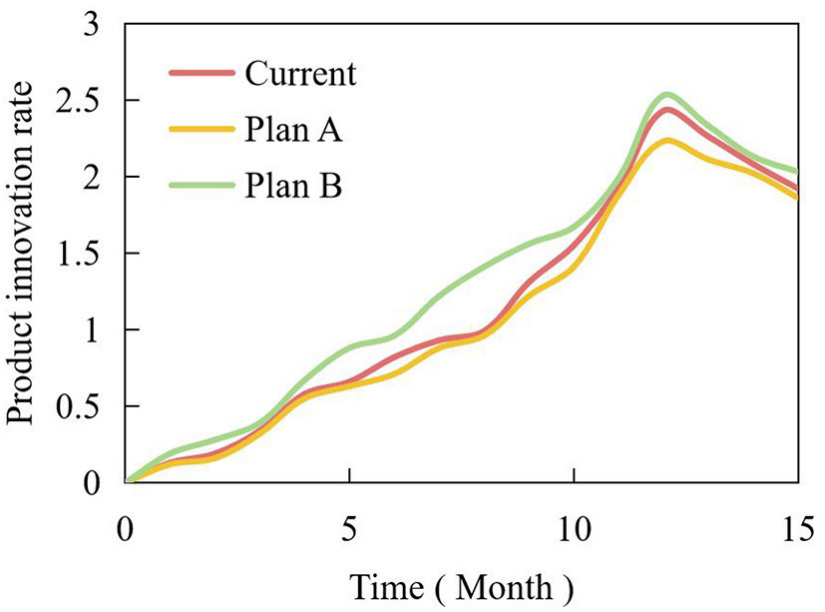
Simulation curves of product innovation rate under different schemes.

**Figure 14:**
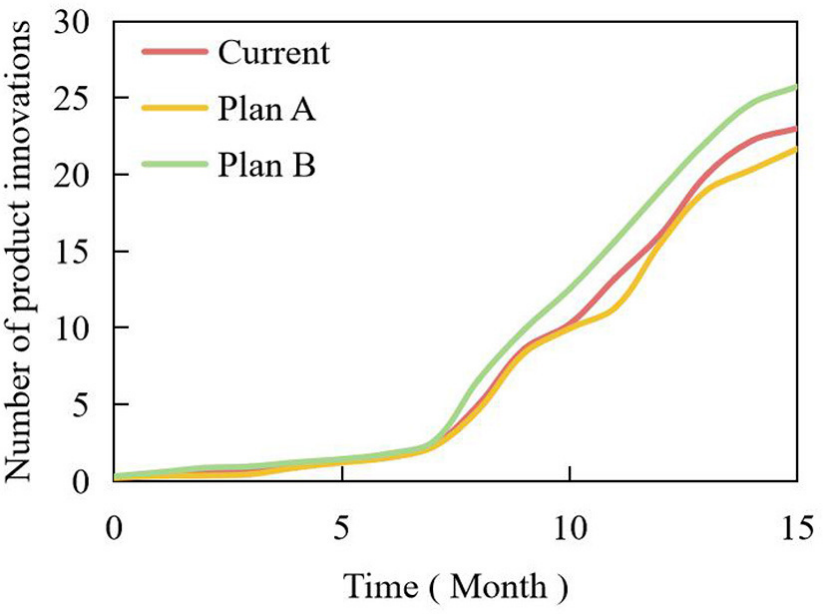
Simulation curves of product innovation numbers under different schemes.

In addition, the influence of consumers' existing knowledge should be considered when branding new products. For discontinuous innovative products, the existing knowledge of consumers is not beneficial, and their understanding of new products, the benefits they proffer to consumers, and their preference for new products are not as good as those of neonates. Additionally, their knowledge of existing product classification affects the consumer's adoption process. Therefore, for continuous innovative products, brand expansion can be carried out because of their strong correlation with the original products; for discontinuous innovative products, because of their low correlation with the original products, new brands should be adopted, and use the opportunity to enter new market segments. Table 2 is the amended plan of process innovation investment. Based on this plan, the simulation curves of product innovation rate and number under different schemes are shown in Figures 13, 14.

**Table 2:** Process innovation input change scheme.

**Plan**	**Innovation**	**Investment ratio of**	**Technological**
	**investment**	**product/process**	**innovation**
		**innovation**	**investment**
Current scheme	23,861	0.2	21,663
Plan A	18,600	0.2	17,824
Plan B	45,000	0.2	27,965

With an increase in process characteristics and further improvement of process complexity of process innovation subsystems, the two indicators of product innovation rate and product innovation number in product innovation subsystem showed an obvious increasing trend. The main reason is that process innovation has greatly improved the technological level of manufacturing enterprises and made the product structure more reasonable. When the elements in the system change, the product, and the process innovation subsystems also change, which makes the interactive relationship between product and process innovation present different modes and characteristics. That is to say, the interactive relationship between product innovation and process innovation is dynamic at different times and in different environments.

With an increase in process characteristics, the subsystem also promotes the development of products in the product innovation subsystem. The innovation quantity of the product and process innovation subsystems increased significantly. The correlation between product and process mainly reflects the correlation between product innovation and process innovation, and this parameter changed with the changes in industries where the manufacturing enterprises are located. Both the product and process innovation subsystems can achieve “changing with constant changes,” so that product and process innovation can interact, promote, and develop harmoniously, and the manufacturing enterprises can be in an invincible position in the face of fierce market competition.

**Figure 15:**
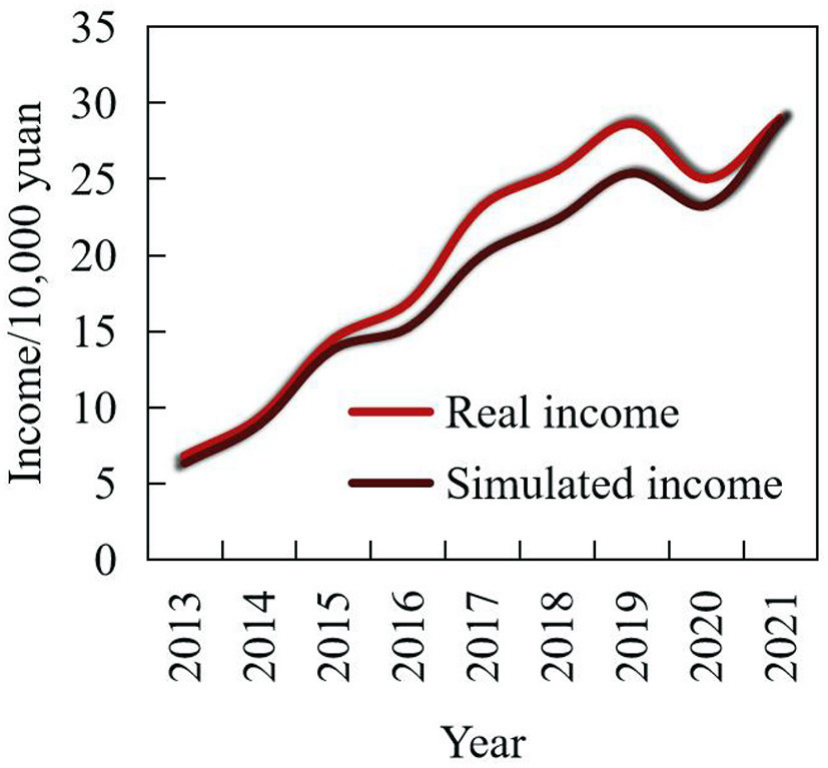
Comparison between actual income and simulated income.

The fundamental purpose of any marketing system is to sell products so that enterprises can make profits. Therefore, the marketing staff of an enterprise should have a systematic understanding of the development trend of enterprise profits and optimize the marketing strategy using simulation. In the above analysis, the new product marketing system was simulated and tested. The development trend of its corporate profits and the simulation results are shown in Figure 15.

An analysis of the enterprises shows the same growth trend as income, cost, and taxes, but there were some minor differences; the profit of the enterprise showed a marginal decreasing trend. This is consistent with the current environment observable in the new product sales market in China. The demand and supply of new products show a strong upward trend, and the sales revenue has been increasing annually, but the sales profit margin has been decreasing and the profitability of enterprises is decreasing.

## Conclusion

This paper established a coupling dynamic model of emotional branding communication in the social media environment. It proposed that when faced with different market types, brands adopt different social marketing strategies and give priority to the marketing intensity and influence frequency. To a certain extent, the interactive mechanism of brand communication discussed in this study will help brands to express their value propositions. They need to maximize the effect of brand communication, improve the quality of products and services, and improve their market competitiveness. This study found that marketing frequency and marketing intensity had different effects on symmetric and asymmetric communications. When facing different types of competitors, weak brands should emphasize their marketing strategy. Through unit consistency test, structure verification test, and effectiveness and rationality test, it was proven that the emotional branding communication model and new product sales interactive simulation model established in this paper were reasonable and effective. It also played a certain role in expressing users' requirements, exchanging utilization experiences, and meeting the needs of the brands.

## Data availability statement

The original contributions presented in the study are included in the article/supplementary material, further inquiries can be directed to the corresponding author/s.

## Ethics statement

Ethical review and approval was not required for the study on human participants in accordance with the local legislation and institutional requirements. Written informed consent from the patients/participants legal guardian/next of kin was not required to participate in this study in accordance with the national legislation and the institutional requirements.

## Author contributions

YZ and ZT: writing and data processing. WZ: literature collection and induction. LH: article typesetting. All authors contributed to the article and approved the submitted version.

## Funding

This work was supported by the National Social Science Fund of China Art Project: Innovative Design Research Based on the Traditional Construction Techniques of Hainan Li Boat Houses (No.: 20BG105); Hainan Provincial Basic and Applied Basic Research Program (Natural Science Field) High-level Talents Project: Research on the Innovative Integration of Medium and Tropical Architecture and Regional Culture in the Construction of Hainan Free Trade Zone (Port) (No.: 2019RC197); Key Project of Education and Teaching Reform research in Hainan Province: Research on curriculum setting and application of four-and-a-half classroom in Hainan Province Junior high and high schools, one school, one product, one specialty, and one multi-functional intangible cultural heritage (No.: Hnjg2020ZD-22).

## Conflict of interest

The authors declare that the research was conducted in the absence of any commercial or financial relationships that could be construed as a potential conflict of interest.

## Publisher's note

All claims expressed in this article are solely those of the authors and do not necessarily represent those of their affiliated organizations, or those of the publisher, the editors and the reviewers. Any product that may be evaluated in this article, or claim that may be made by its manufacturer, is not guaranteed or endorsed by the publisher.
